# Supplementary material for: Comparison of herpes simplex virus 1 genomic diversity between adult sexual transmission partners with genital infection
Source: PLoS Pathog. 2022 May 19;18(5):e1010437. doi: 10.1371/journal.ppat.1010437 (PMC9119503; doi:10.1371/journal.ppat.1010437)
Supplement: S1 Table — This file contains a list of strain names, GenBank accession numbers, and references for the previously published 65 HSV-1 genomes used in the network graph analysis in Fig 2. (PDF) [file ppat.1010437.s001.pdf]

S1 Table: List of previously published HSV-1 genomes used for phylogenetic analyses

Virus Isolate	Country (with location detail, if available)	GenBank Accession #	References
SC16	Spain (Madrid)	KX946970	[1]
172/2010	Germany (Jena)	LT594105	[2]
2158/2007	Germany (Jena)	LT594106	[2]
3083/2008	Germany (Jena)	LT594107	[2]
1319/2005	Germany	LT594108	[2]
270/2007	Germany (Manebach)	LT594109	[2]
66/2007	Germany (Jena)	LT594110	[2]
1394/2005	Germany	LT594111	[2]
369/2007	Germany (Jena)	LT594112	[2]
160/1982	Germany (Erfurt)	LT594192	[2]
132/1998	Germany (Gelsenkirchen)	LT594457	[2]
L2	Russia (Moscow)	KT780616	[3]
KOS79	U.S.A. (Madison, WI)	KT425109	[4]
HSV-1/0116209/India/2011	India (Pune)	KJ847330	[5]
H166	U.S.A.	KM222726	[6]
H166syn	U.S.A.	KM222727	[6]
17	U.K. (Glasgow)	JN555585	[7,8]
CR38	China (Shenyang)	HM585508	[9]
E06	Kenya (Nairobi)	HM585496	[9]
E07	Kenya (Nairobi)	HM585497	[9]
E08	Kenya (Nairobi)	HM585498	[9]
E10	Kenya (Nairobi)	HM585499	[9]
E11	Kenya (Nairobi)	HM585500	[9]
E12	Kenya (Nairobi)	HM585501	[9]
E13	Kenya (Nairobi)	HM585502	[9]
E14	Kenya (Nairobi)	HM585510	[9]
E15	Kenya (Nairobi)	HM585503	[9]
E19	Kenya (Nairobi)	HM585511	[9]
E22	Kenya (Nairobi)	HM585504	[9]
E23	Kenya (Nairobi)	HM585505	[9]
E25	Kenya (Nairobi)	HM585506	[9]
E35	Kenya (Nairobi)	HM585507	[9]
R11	South Korea (Seoul)	HM585514	[9]
R62	South Korea (Seoul)	HM585515	[9]
S23	Japan (Sapporo)	HM585512	[9]
S25	Japan (Sapporo)	HM585513	[9]
F	U.S.A. (Chicago, IL)	GU734771	[10,11]
H129	U.S.A. (San Francisco, CA)	GU734772	[10,12]
McKrae	U.S.A. (Gainesville, FL)	JQ730035, JX142173	[13–15]
KOS	U.S.A. (Houston, TX)	JQ673480, JQ780693	[16,17]
HF10	U.S.A. (New York, NY)	DQ889502	[18]
Ty25	Japan (Tokyo)	MH999840	[19]
Ty148	Japan (Tokyo)	MH999841	[19]
K86	Japan (Osaka)	MH999839	[19]

Virus Isolate	Country (with location detail, if available)	GenBank Accession #	References
K47	Japan (Osaka)	MH999838	[19]
KOS63	U.S.A. (Houston, TX)	KT425110	[4]
134	U.S.A. (Seattle, WA)	JN400093	[20]
CJ311	U.S.A. (Seattle, WA)	JN420338	[20]
CJ970	U.S.A. (Seattle, WA)	JN420341	[20]
v.29	U.S.A. (Seattle, WA)	MH102298	[21]
Neonate_disseminated_blood	U.S.A. (Iowa City, Iowa)	MK952184	[22]
Mother_viremic_blood	U.S.A. (Iowa City, Iowa)	MK952185	[22]
MacIntyre	Berkeley, CA	KM222720	[23]
N-7	U.S.A. (Cincinnati, OH)	KY922719	[24]
R-13	U.S.A. (Cincinnati, OH)	KY922718	[24]
H1412	Finland	MH999851	[25]
H15119	Finland	MH999850	[25]
H12118	Finland	MH999847	[25]
H12113	Finland	MH999842	[25]
H12114	Finland	MH999844	[25]
H12117	Finland	MH999845	[25]
H1211	Finland	MH999843	[25]
H1215	Finland	MH999846	[25]
H1311	Finland	MH999848	[25]
H1312	Finland	MH 999849	[25]
v40_nd_gen	U.S.A. (Seattle, WA)	ON007132	Present study
v40_y14_oral1	U.S.A. (Seattle, WA)	ON007153	Present study
v40_y14_oral2	U.S.A. (Seattle, WA)	ON007154	Present study
v41_d100_gen	U.S.A. (Seattle, WA)	ON007139	Present study
v41_d354_oral	U.S.A. (Seattle, WA)	ON007157	Present study
v43_d130_gen_les	U.S.A. (Seattle, WA)	ON007160	Present study
v43_d131_gen_les	U.S.A. (Seattle, WA)	ON007137	Present study
v43_d338_gen_les	U.S.A. (Seattle, WA)	ON007138	Present study
v43_d339_gen	U.S.A. (Seattle, WA)	ON007164	Present study
v42_d17_gen_les	U.S.A. (Seattle, WA)	ON007141	Present study
v42_d137_gen_les	U.S.A. (Seattle, WA)	ON007156	Present study
v42_d349_gen	U.S.A. (Seattle, WA)	ON007148	Present study
v42_d349_gen_les	U.S.A. (Seattle, WA)	ON007147	Present study
v42_d350_gen	U.S.A. (Seattle, WA)	ON007152	Present study
v42_d350_gen_les	U.S.A. (Seattle, WA)	ON007159	Present study
v42_d352_gen	U.S.A. (Seattle, WA)	ON007135	Present study
v42_d352_gen_les	U.S.A. (Seattle, WA)	ON007162	Present study
v44_d2_gen_les	U.S.A. (Seattle, WA)	ON007134	Present study
v45_d4_gen_les	U.S.A. (Seattle, WA)	ON007145	Present study
v46_d345_gen	U.S.A. (Seattle, WA)	ON007144	Present study
v46_y16_oral	U.S.A. (Seattle, WA)	ON007133	Present study
v46_d348_gen	U.S.A. (Seattle, WA)	ON007150	Present study
v46_d349_gen	U.S.A. (Seattle, WA)	ON007146	Present study
v46_d349_gen_les1	U.S.A. (Seattle, WA)	ON007158	Present study
v46_d349_gen_les2	U.S.A. (Seattle, WA)	ON007143	Present study
v46_d349_gen_les3	U.S.A. (Seattle, WA)	ON007151	Present study

Virus Isolate	Country (with location detail, if available)	GenBank Accession #	References
v47_d61-79_gen	U.S.A. (Seattle, WA)	ON007163	Present study
v48_d100_oral	U.S.A. (Seattle, WA)	ON007155	Present study
v48_d101_oral	U.S.A. (Seattle, WA)	ON007136	Present study
v48_d102_oral	U.S.A. (Seattle, WA)	ON007161	Present study
v48_d103_oral	U.S.A. (Seattle, WA)	ON007140	Present study
v49_d257_gen_les	U.S.A. (Seattle, WA)	ON007142	Present study
v49_d349_gen_les	U.S.A. (Seattle, WA)	ON007149	Present study

References

1. Rastrojo A, López-Muñoz AD, Alcamí A. Genome Sequence of Herpes Simplex Virus 1 Strain SC16. Genome Announc. 2017;5: e01392-16. doi:10.1128/genomeA.01392-16
2. Pfaff F, Groth M, Sauerbrei A, Zell R. Genotyping of herpes simplex virus type 1 (HSV-1) by whole genome sequencing. J Gen Virol. 2016. doi:10.1099/jgv.0.000589
3. Skoblov MYu, Lavrov AV, Bragin AG, Zubtsov DA, Andronova VL, Galegov GA, et al. The genome nucleotide sequence of herpes simplex virus 1 strain L2. Russ J Bioorganic Chem. 2017;43: 140–142. doi:10.1134/S1068162016060133
4. Bowen CD, Renner DW, Shreve JT, Tafuri Y, Payne KM, Dix RD, et al. Viral forensic genomics reveals the relatedness of classic herpes simplex virus strains KOS, KOS63, and KOS79. Virology. 2016;492: 179–186. doi:10.1016/j.virol.2016.02.013
5. Bondre VP, Sankararaman V, Andhare V, Tupekar M, Sapkal GN. Genetic characterization of human herpesvirus type 1: Full-length genome sequence of strain obtained from an encephalitis case from India. Indian J Med Res. 2016;144: 750–760. doi:10.4103/ijmr.IJMR_747_14
6. Parsons LR, Tafuri YR, Shreve JT, Bowen CD, Shipley MM, Enquist LW, et al. Rapid Genome Assembly and Comparison Decode Intrastrain Variation in Human Alphaherpesviruses. mBio. 2015;6: e02213-14. doi:10.1128/mBio.02213-14
7. McGeoch DJ, Dalrymple MA, Davison AJ, Dolan A, Frame MC, McNab D, et al. The complete DNA sequence of the long unique region in the genome of herpes simplex virus type 1. J Gen Virol. 1988;69: 1531–74.
8. McGeoch DJ, Dolan A, Donald S, Rixon FJ. Sequence determination and genetic content of the short unique region in the genome of herpes simplex virus type 1. J Mol Biol. 1985;181: 1–13. doi:10.1016/0022-2836(85)90320-1
9. Szpara ML, Gatherer D, Ochoa A, Greenbaum B, Dolan A, Bowden RJ, et al. Evolution and diversity in human herpes simplex virus genomes. J Virol. 2014;88: 1209–27. doi:10.1128/JVI.01987-13
10. Szpara ML, Parsons L, Enquist LW. Sequence variability in clinical and laboratory isolates of herpes simplex virus 1 reveals new mutations. J Virol. 2010;84: 5303–13. doi:10.1128/JVI.00312-10
11. Ejercito PM, Kieff ED, Roizman B. Characterization of herpes simplex virus strains differing in their effects on social behaviour of infected cells. J Gen Virol. 1968;2: 357–364.

12. Dix RD, McKendall RR, Baringer JR. Comparative neurovirulence of herpes simplex virus type 1 strains after peripheral or intracerebral inoculation of BALB/c mice. Infect Immun. 1983;40: 103–112.

13. Watson G, Xu W, Reed A, Babra B, Putman T, Wick E, et al. Sequence and comparative analysis of the genome of HSV-1 strain McKrae. Virology. 2012;433: 528–37. doi:10.1016/j.virol.2012.08.043

14. Macdonald SJ, Mostafa HH, Morrison LA, Davido DJ. Genome sequence of herpes simplex virus 1 strain McKrae. J Virol. 2012;86: 9540–9541. doi:10.1128/JVI.01469-12

15. Williams LE, Nesburn AB, Kaufman HE. Experimental induction of disciform keratitis. Arch Ophthalmol. 1965;73: 112–114. doi:10.1001/archopht.1965.00970030114023

16. Macdonald SJ, Mostafa HH, Morrison LA, Davido DJ. Genome sequence of herpes simplex virus 1 strain KOS. J Virol. 2012;86: 6371–6372. doi:10.1128/JVI.00646-12

17. Smith KO. Relationship Between the Envelope and the Infectivity of Herpes Simplex Virus. Exp Biol Med. 1964;115: 814–816. doi:10.3181/00379727-115-29045

18. Ushijima Y, Luo C, Goshima F, Yamauchi Y, Kimura H, Nishiyama Y. Determination and analysis of the DNA sequence of highly attenuated herpes simplex virus type 1 mutant HF10, a potential oncolytic virus. Microbes Infect. 2007;9: 142–149. doi:10.1016/j.micinf.2006.10.019

19. Umene K, Yoshida M. Genomic characterization of two predominant genotypes of herpes simplex virus type 1. Arch Virol. 1993;131: 29–46.

20. Kolb AW, Adams M, Cabot EL, Craven M, Brandt CR. Multiplex sequencing of seven ocular herpes simplex virus type-1 genomes: phylogeny, sequence variability, and SNP distribution. Invest Ophthalmol Vis Sci. 2011;52: 9061–73. doi:10.1167/iovs.11-7812

21. Shipley MM, Renner DW, Ott M, Bloom DC, Koelle DM, Johnston C, et al. Genome-wide surveillance of genital herpes simplex virus type 1 from multiple anatomic sites over time. J Infect Dis. 2018;218: 595–605. doi:10.1093/infdis/jiy216

22. Shipley MM, Renner DW, Pandey U, Ford B, Bloom DC, Grose C, et al. Personalized viral genomic investigation of herpes simplex virus 1 perinatal viremic transmission with dual fatality. Mol Case Stud. 2019;5: a004382. doi:10.1101/mcs.a004382

23. Szpara ML, Tafuri YR, Parsons L, Shreve JT, Engel EA, Enquist LW. Genome sequence of the anterograde-spread-defective herpes simplex virus 1 strain MacIntyre. Genome Announc. 2014;2. doi:10.1128/genomeA.01161-14

24. Pandey U, Renner DW, Thompson RL, Szpara ML, Sawtell NM. Inferred father-to-son transmission of herpes simplex virus results in near-perfect preservation of viral genome identity and in vivo phenotypes. Sci Rep. 2017;7: 13666. doi:10.1038/s41598-017-13936-6

25. Bowen CD, Paavilainen H, Renner DW, Palomäki J, Lehtinen J, Vuorinen T, et al. Comparison of herpes simplex virus 1 strains circulating in Finland demonstrates the uncoupling of whole-genome relatedness and phenotypic outcomes of viral infection. Longnecker RM, editor. J Virol. 2019;93: e01824-18. doi:10.1128/JVI.01824-18
