# Supplementary material for: Comparison of herpes simplex virus 1 genomic diversity between adult sexual transmission partners with genital infection
Source: PLoS Pathog. 2022 May 19;18(5):e1010437. doi: 10.1371/journal.ppat.1010437 (PMC9119503; doi:10.1371/journal.ppat.1010437)
Supplement: Attachment — Submitted filename: Response_to_Review_PLoS_Path_transmission_paper_v7.pdf [file ppat.1010437.s003.pdf]

We appreciate the suggestions of the Editor and Reviewers and have incorporated these into the revised text. In addition to addressing the Reviewers' suggestions point-by-point below, we have made an effort to eliminate jargon in the phrasing of the manuscript and its conclusions. The Editor's and Reviewers' specific comments are included below, in *blue italics*. **Page and line numbers below refer to the Marked-up Manuscript file**, which highlights all changes from the originally submitted version. We appreciate the contributions of the review process and look forward to hearing back from you.

Editorial Comment

In addition, to engage a broader PLOS Pathogens readership, please, restate your major conclusions throughout the manuscript avoiding jargon.

We have adjusted the text throughout the manuscript to reduce the use of jargon and improve clarity. This included simplifying clinical terms such as 'anogenital' and 'orolabial' to 'genital' and 'oral', and clarifying our use of descriptors such as 'source' and 'recipient'. These terms were simplified both in the text and in Figure labels.

Reviewer's Responses to Questions

Part I - Summary

Reviewer #1: Both herpes simplex virus 1 and 2 (HSV-1 and HSV-2) can be responsible for genital herpes lesions. HSV-1 classically oral infections and HSV-2 genital infections, however HSV-1 epidemiology is evolving in favour of an increasing number of primary infections occur in the (ana-)genital anatomical niche. Viruses could adapt to their host over time thanks to genetic adaptation. As few data are known for HSV, especially for HSV-1 in the context of genital herpes, all studies concerning this subject are quite interesting. Then, the authors, Rathbun at al., try to study viral genetic diversity in the context of HSV-1 genital herpes. Indeed, they used deep-sequencing analysis to characterize the consensus HSV-1 sequences and to detect minor variants (MVs) within paired sexual partner samples.

Reviewer #2: Rathbun et al describe an important and elegant study of genetic variation in herpesvirus genomes among recently infected sexual partners. Both the study and the manuscript are highly accomplished and the authors are to be congratulated.

We appreciate both Reviewers' summaries and esteem for the study.

Part II – Major Issues: Key Experiments Required for Acceptance

Reviewer #1: I have read through the manuscript and I have major comments.

- To my opinion, the most bothering fault of this study is the analysis of sequencing data. Indeed how is it possible to take into account sequencing data whereas average coverage is very low (see Table 2: e.g. Sample v47_d61-79_gen with 31x of average coverage associated with low viral load of 2.6 cp/mL of virus as data was obtained form 15000 reads with pooled from two replicates or Sample v41_d354_oral with 28x of average coverage associated with low viral load of 2.2 cp/mL of virus as data was obtained form 4100 reads). The use of threshold of 20% supposedly more stringent is not appropriated. To my opinion, to be relevant, only samples with coverage depth <100x should not be considered. Most of the time, low average coverage is associated few reads available and high MV detection: e.g. Sample v47_d61-79_gen with 31x of average coverage associated with 225 MVs or Sample v41_d354_oral with 28x of average coverage associated with 144 MVs. Can we trust that?

The challenge for this and similar studies that employ direct-from-patient sequencing of viral genetic material is that we cannot always obtain as high a coverage depth as we would like. This situation is more

pervasive for chronic viral pathogens such as herpesviruses, where the overall viral load during episodes of reactivation can be quite low [1–4], unless the study population is immunocompromised [5–9]. Because episodes of spontaneous viral reactivation are relatively infrequent, we chose to maximize data availability and to include all samples that were successfully sequenced in our study. We convey to the reader the differences in coverage depth to make them aware of these distinctions, but we do not find it useful to exclude data from episodes of sparse shedding. **As technologies for viral capture and deep sequencing improve, we anticipate that others will build upon these initial studies and expand our understanding of how viral diversity changes during episodes of high vs. low viral shedding.** As such, these initial insights are highly valuable for indicating areas of interest for future studies. We have added text to the results to highlight the differences in coverage depth more clearly (e.g. Lines 230-233, 410-411, 421, 448-449, 466-496, 1287-1290, 1308-1309, and 1324-1325 in the Marked-Up Manuscript).

As indicated in our reply to Reviewer 1's next point, our use of stringent quality-filtering criteria also helps to ensure that even low-coverage samples have high-quality data. In addition to the quality-trimming filters noted in the next reply-point below (Lines 907-910), our analyses of minor variants (MV) include additional quality-controls. These included de-duplication of reads, a minimum coverage depth, and elimination of any MVs with evidence of strand bias or localization near homopolymers (Lines 940-951). These filters meet and in most cases exceed the stringency of those used in comparable studies of HSV, VZV, and other herpesvirus genomic diversity [7,10–13], lending strength to their inclusion in this dataset. We include below an example screen-shot of the sequence-read pileup used for minor variant detection in a low-coverage oral sample from participant 41 (v41_d354_oral), to demonstrate the "clean" appearance of the variants that pass this quality filtering (i.e., these minor variants are not in areas of gapped coverage, mis-alignment, or repetitive regions).

Example alignment from v41_d354_oral

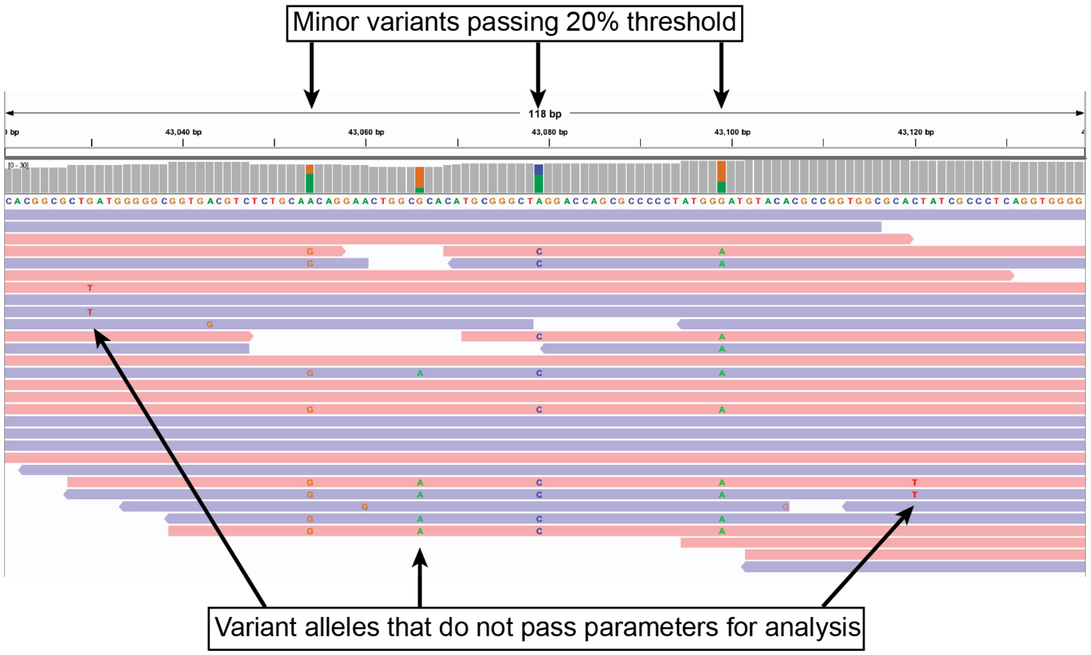

There are five samples with an average coverage less than 100X in our analysis, of which two had zero MVs, while the other three had 5-530 minor variants. In the first submission of our manuscript, we correctly noted that the sequencing libraries for these samples had been run twice on our MiSeq to increase their coverage. However the second round of sequencing data had not yet been incorporated into our analyses of minor variants. We have now corrected this omission and revised Table 2 accordingly. The additional sequence depth for MV analysis led to an increase in the number of minor variants for several samples. For example, the sample v41_d354_oral (28X coverage depth) went from 144 MVs to a total of 530 MVs detected. A similar increase in MV count (225 to 257) was detected for v47_d61-79_gen (31X coverage depth). Other

samples such as v48_d102 and d103_oral did not show any change in MV count. Only one sample (v46_y16_oral) showed a decrease in MV count, although these variants remained present in the sample, just below the 20% threshold for detection. The positions of these minor variants in the v46 sample still match those observed in their transmission partner v47 (Figure 7, Supporting Table 2). As we noted in the text (Lines 421-423), *"…variants that are detected and shared in both partners are notable and unlikely to occur in these samples by chance."* These observations suggests that our conservative threshold of 20% is sufficient for analysis of MVs from samples with <100X average coverage, and that higher coverage data do not lead to a reduced detection of minor variants – instead the level of minor variant diversity appears to differ by anatomical niche, sampling site (e.g. a wide area vs. lesion-specific), and timing of sample collection. These points are reflected in the revised text of the discussion (Lines 542-543, 671-679, 686-687, 730-760, and 773-777 of the Marked-Up Manuscript).

It is interesting to note that 5 of the 6 samples with the highest levels of minor variants are derived from the oral niche. Although this study was not designed to focus on HSV-1 oral shedding, it is possible that either the oral niche, or the long duration of those pre-existing oral infections (vs. the new primary genital infections that were the focus of this study), may have contributed to this outcome (see Lines 773-777). In a prior study that included 9 surface swabs (oral, ocular, genital, or skin) as control samples for genome-wide MV analysis, the authors found comparable levels of MVs (10-25) in most samples, except for one genital swab (from a lesion in a 65 year old individual) that contained ~150 minor variants [14]. The authors speculated that super-infection and recombination may have led to the high level of MVs detected in that sample [14]. Given the 3.5-fold larger number of samples sequenced here, we find that our data are comparable and suggest that future studies should explore distinct niches, multiple time points, and multiple reactivation episodes per person, at different stages of infection (primary vs. multi-year infections) to discern the extent and frequency of MV diversity in each infection. We have added text to the discussion to reflect these points (Lines 646-648, 671-675, 684-687, 720-724 in the Marked-Up Manuscript).

Moreover, the authors do not discuss quality criteria for sequence trimming in the MM section. May the authors cite publications where these criteria have been used for NGS analysis for HSV genomic diversity estimation?

In our materials and methods we briefly summarized our quality-trimming approach, which we have previously documented [15]. In our revised manuscript, we now define these parameters more explicitly (Lines 907-910): "*…reads were screened using Trimmomatic [16] to remove artifacts and any Illumina adapter sequences, and trimmed to remove low quality bases (minimum PHRED score 30, over a 15 bp window size). This process also removes any short read fragments (minimum size 30 bp) and any unpaired reads."* The same quality-filtering parameters have been used in all of our previous studies of HSV-1 and HSV-2 genomic diversity (e.g. [17–22]) and mirror those used for VZV [10,11].

Our choice of quality criteria is based on those of other leading groups in the broader field of herpesvirus genomics. For example, Suarez and colleagues [12] use the comparable program TrimGalore for quality trimming, with a Phred quality score of 10. In our analysis, we require Phred quality score of 30, which indicates a 0.1% chance of an incorrect base call – as opposed to a Phred score of 10, which equates to a 10% chance that the base call is incorrect. By using Phred 30 as our quality parameter, we maximize the quality of the coverage that we obtain [23] and have confidence in the variant calls observed in these data.

- According to the authors, detection of MVs within the viral population revealed both shared and unique patterns of genetic diversity between partners, and between anatomical niches. But how to make the clear distinction anal and genital niche with self-sampling samples.

In comparing minor variants (MVs) in different viral populations, our main focus in anatomical niche comparisons was on the oral vs. the genital niche. The manner of sample collection in this study – which involved patients self-swabbing the entire anogenital area from front to back – would make it impossible to

distinguish between genital vs. anal shedding in the self-collected samples. Prior work from co-authors Johnston and Szpara has included spatially-segregated sampling of the genital area, and those studies demonstrated that genital and anal shedding sites can be distinct [3,24]. However that was not the focus of the study here. We have clarified the text to better note this distinction (Lines 166-169, 543, 671-675, 684-687, and 829-836). In keeping with the editorial request to reduce jargon, we have also limited our use of the term "anogenital" to the Methods (i.e. to define the sample collection area) and we now use the term "genital" to refer to these samples.

- Additionally, the authors discuss that genetic drift was detected from spatiotemporally separated samples in as little as three days. This period of time of 3 days appear to be unrealistic. Indeed, even under antiviral selection pressure for instance the evolution pf HSV-1 do not reach such a level of MV within viral genes implicated in antiviral resistance.

There is a substantial body of literature investigating the ability of both HSV-1 and HSV-2 to spontaneously generate acyclovir- (ACV) and other antiviral-drug resistance in a matter of days [25–28]. These assays generally involve exposing clinical isolates from drug-naïve individuals to ACV, and measuring the number of ACV-resistant plaques that arise in 2-3 days, or comparing the titer produced by plating a given isolate with or without ACV present [25–28]. Based on this well-established precedent, we do not find it surprising to see fluctuations at the minor variant level over this short of a time scale (i.e. a few days). In fact, the low frequency of the minor variant alleles that we observed mirrors the findings of these prior antiviral-resistance studies, which used ACV-sensitive clinical isolates as the starting point for observing the rapid "evolution" of antiviral resistance. Using different *in vitro* selection models, these prior studies demonstrated that antiviral resistance can occur either by rapid selection for previously existing rare alleles (i.e. minor variants) (e.g. [25]), or by the spontaneous generation and rapid selection of new alleles (e.g. [26]). In sampling MVs from human shedding, we can only note the presence of MVs when they are detected. In samples where MVs are not detected, we cannot differentiate the disappearance of these rare alleles from the population, vs. the possibility that these rare alleles have simply dropped below our threshold for detection. The latent neuronal reservoir of HSV genomes represents another location where these rare alleles may reside, from which they could rapidly appear upon neuronal reactivation and spread into surface epithelial cells. We have summarized these ideas in a recent review article [29], and we have now added these points to the discussion as well. We have also highlighted a prior published example of rapid changes in minor variant frequency, which were observed over three days of shedding in a patient with a long-running genital infection [24], which provides another precedent for this rate of MV change (Lines 646-648 in the Marked-Up Manuscript).

- It appears there is some discordance between text/tables/figure data makes difficult the interpretation of the study. Either I do not understand or this formulation is not clear enough for well-understanding! I think the authors simplify the tables and the figures to get straight to the point.

As noted by Reviewer 2 below, there were several places in the manuscript where a reference to one of the Tables was missing, or the reference to a figure was incorrect (e.g. pointing to Fig 6B instead of 6C). We appreciate both Reviewers bringing this to our attention, and we have now checked and corrected all references to Figures and Tables. We have also added or revised our phrasing to clarify the point of the data referenced in figures and tables (e.g. Lines 166-169, 673-675, 711-713, 1293-1294).

Reviewer #2: (No Response)

Part III – Minor Issues: Editorial and Data Presentation Modifications

Reviewer #1: NONE

Reviewer #2: There are a number of places where it appears the text is referring to the wrong figure number or sub-number e.g. 6b rather than 6 C on page 13 line 292. It would be useful for the authors to systematically review all such references for accuracy.

We apologize and appreciate the Reviewer bringing these to our attention. All references to figures and tables have been checked and corrected where necessary.

The discussion should include comments on the limitations of the methodology in terms of variation in the depth of sequencing coverage between samples and the implications of this on the ability to detect MV.

As noted in our response to Reviewer 1 above, we have added text to the Results highlight the differences in coverage depth and to discuss how conservative approaches to minor variant detection mitigate these limitations (e.g. Lines 230-233 and 466-496). We have also added Supporting Table 2, which provides positional data and quantitative statistics for all MV detected in these samples. These data allow the reader to compare MV position and frequency within and between transmission pairs. Finally, we have expanded the Discussion to note how future studies can employ diverse sampling strategies to better capture the transient nature of HSV-1 viral population diversity in human infections (Lines 673-679, 686-687, 689-692, 773-777, 792-793).

References

1. Lafferty WE, Coombs RW, Benedetti J, Critchlow C, Corey L. Recurrences after Oral and Genital Herpes Simplex Virus Infection. N Engl J Med. 1987;316: 1444–1449. doi:10.1056/NEJM198706043162304
2. Engelberg R, Carrell D, Krantz E, Corey L, Wald A. Natural history of genital herpes simplex virus type 1 infection. Sex Transm Dis. 2003;30: 174–177.
3. Tronstein E, Johnston C, Huang M-L, Selke S, Magaret A, Warren T, et al. Genital shedding of herpes simplex virus among symptomatic and asymptomatic persons with HSV-2 infection. Jama. 2011;305: 1441–1449.
4. Ramchandani M, Kong M, Tronstein E, Selke S, Mikhaylova A, Magaret A, et al. Herpes Simplex Virus Type 1 Shedding in Tears and Nasal and Oral Mucosa of Healthy Adults: Sex Transm Dis. 2016;43: 756–760. doi:10.1097/OLQ.0000000000000522
5. Kim HN, Meier A, Huang M-L, Kuntz S, Selke S, Celum C, et al. Oral Herpes Simplex Virus Type 2 Reactivation in HIV-Positive and -Negative Men. J Infect Dis. 2006;194: 420–427. doi:10.1086/505879
6. Houldcroft CJ, Bryant JM, Depledge DP, Margetts BK, Simmonds J, Nicolaou S, et al. Detection of Low Frequency Multi-Drug Resistance and Novel Putative Maribavir Resistance in Immunocompromised Pediatric Patients with Cytomegalovirus. Front Microbiol. 2016;7. doi:10.3389/fmicb.2016.01317
7. Hage E, Wilkie GS, Linnenweber-Held S, Dhingra A, Suárez NM, Schmidt JJ, et al. Characterization of Human Cytomegalovirus Genome Diversity in Immunocompromised Hosts by Whole-Genome Sequencing Directly From Clinical Specimens. J Infect Dis. 2017;215: 1673–1683. doi:10.1093/infdis/jix157
8. Cudini J, Roy S, Houldcroft CJ, Bryant JM, Depledge DP, Tutill H, et al. Human cytomegalovirus haplotype reconstruction reveals high diversity due to superinfection and evidence of within-host recombination. Proc Natl Acad Sci. 2019; 201818130. doi:10.1073/pnas.1818130116
9. Pang J, Slyker JA, Roy S, Bryant J, Atkinson C, Cudini J, et al. Mixed cytomegalovirus genotypes in HIV-positive mothers show compartmentalization and distinct patterns of transmission to infants. eLife. 2020;9: e63199. doi:10.7554/eLife.63199
10. Depledge DP, Palser AL, Watson SJ, Lai IY-C, Gray ER, Grant P, et al. Specific Capture and Whole-Genome Sequencing of Viruses from Clinical Samples. Jhaveri R, editor. PLoS ONE. 2011;6: e27805. doi:10.1371/journal.pone.0027805
11. Depledge DP, Yamanishi K, Gomi Y, Gershon AA, Breuer J. Deep Sequencing of Distinct Preparations of the Live Attenuated Varicella-Zoster Virus Vaccine Reveals a Conserved Core of Attenuating Single-Nucleotide Polymorphisms. Frueh K, editor. J Virol. 2016;90: 8698–8704. doi:10.1128/JVI.00998-16

12. Suárez NM, Wilkie GS, Hage E, Camiolo S, Holton M, Hughes J, et al. Human Cytomegalovirus Genomes Sequenced Directly From Clinical Material: Variation, Multiple-Strain Infection, Recombination, and Gene Loss. J Infect Dis. 2019;220: 781–791. doi:10.1093/infdis/jiz208
13. Suárez NM, Musonda KG, Escriva E, Njenga M, Agbueze A, Camiolo S, et al. Multiple-Strain Infections of Human Cytomegalovirus With High Genomic Diversity Are Common in Breast Milk From Human Immunodeficiency Virus–Infected Women in Zambia. J Infect Dis. 2019;220: 792–801. doi:10.1093/infdis/jiz209
14. Lassalle F, Beale MA, Bharucha T, Williams CA, Williams RJ, Cudini J, et al. Whole genome sequencing of Herpes Simplex Virus 1 directly from human cerebrospinal fluid reveals selective constraints in neurotropic viruses. Virus Evol. 2020;6. doi:10.1093/ve/veaa012
15. Parsons LR, Tafuri YR, Shreve JT, Bowen CD, Shipley MM, Enquist LW, et al. Rapid genome assembly and comparison decode intrastrain variation in human alphaherpesviruses. mBio. 2015;6: e02213-14. doi:10.1128/mBio.02213-14
16. Bolger AM, Lohse M, Usadel B. Trimmomatic: a flexible trimmer for Illumina sequence data. Bioinformatics. 2014;30: 2114–2120. doi:10.1093/bioinformatics/btu170
17. Bowen CD, Renner DW, Shreve JT, Tafuri Y, Payne KM, Dix RD, et al. Viral forensic genomics reveals the relatedness of classic herpes simplex virus strains KOS, KOS63, and KOS79. Virology. 2016;492: 179–186. doi:10.1016/j.virol.2016.02.013
18. Pandey U, Bell AS, Renner DW, Kennedy DA, Shreve JT, Cairns CL, et al. DNA from dust: Comparative genomics of large DNA viruses in field surveillance samples. Smith GA, editor. mSphere. 2016;1: e00132-16. doi:10.1128/mSphere.00132-16
19. Shipley MM, Renner DW, Pandey U, Ford B, Bloom DC, Grose C, et al. Personalized viral genomic investigation of herpes simplex virus 1 perinatal viremic transmission with dual fatality. Mol Case Stud. 2019;5: a004382. doi:10.1101/mcs.a004382
20. Bowen CD, Paavilainen H, Renner DW, Palomäki J, Lehtinen J, Vuorinen T, et al. Comparison of herpes simplex virus 1 strains circulating in Finland demonstrates the uncoupling of whole-genome relatedness and phenotypic outcomes of viral infection. Longnecker RM, editor. J Virol. 2019;93: e01824-18. doi:10.1128/JVI.01824-18
21. Akhtar LN, Bowen CD, Renner DW, Pandey U, Della Fera AN, Kimberlin DW, et al. Genotypic and Phenotypic Diversity of Herpes Simplex Virus 2 within the Infected Neonatal Population. Goodrum F, editor. mSphere. 2019;4: e00590-18. doi:10.1128/mSphere.00590-18
22. Kuny CV, Bowen CD, Renner DW, Johnston CM, Szpara ML. In vitro evolution of herpes simplex virus 1 (HSV-1) reveals selection for syncytia and other minor variants in cell culture. Virus Evol. 2020;6. doi:10.1093/ve/veaa013
23. Illingworth CJR, Roy S, Beale MA, Tutill H, Williams R, Breuer J. On the effective depth of viral sequence data. Virus Evol. 2017;3. doi:10.1093/ve/vex030
24. Shipley MM, Renner DW, Ott M, Bloom DC, Koelle DM, Johnston C, et al. Genome-wide surveillance of genital herpes simplex virus type 1 from multiple anatomic sites over time. J Infect Dis. 2018;218: 595–605. doi:10.1093/infdis/jiy216
25. Parris DS, Harrington JE. Herpes simplex virus variants resistant to high concentrations of acyclovir exist in clinical isolates. Antimicrob Agents Chemother. 1982;22: 71–77.
26. Hall JD, Coen DM, Fisher BL, Weisslitz M, Randall S, Almy RE, et al. Generation of genetic diversity in herpes simplex virus: An antimutator phenotype maps to the DNA polymerase locus. Virology. 1984;132: 26–37. doi:10.1016/0042-6822(84)90088-6
27. Sarisky RT, Nguyen TT, Duffy KE, Wittrock RJ, Leary JJ. Difference in incidence of spontaneous mutations between Herpes simplex virus types 1 and 2. Antimicrob Agents Chemother. 2000;44: 1524–1529.
28. Shin YK, Cai G-Y, Weinberg A, Leary JJ, Levin MJ. Frequency of Acyclovir-Resistant Herpes Simplex Virus in Clinical Specimens and Laboratory Isolates. J Clin Microbiol. 2001;39: 913–917. doi:10.1128/JCM.39.3.913-917.2001
29. Rathbun MM, Szpara ML. A holistic perspective on herpes simplex virus (HSV) ecology and evolution. Advances in Virus Research. Elsevier; 2021. pp. 27–57. doi:10.1016/bs.aivir.2021.05.001
